# IBA-1^+^CD68^+^ Germinal Center Macrophages Harbor Proviral and Inducible Clade C HIV Reservoirs in ART-Suppressed Human Lymph Nodes

**DOI:** 10.21203/rs.3.rs-8406328/v1

**Published:** 2026-01-07

**Authors:** Merantha Moodley, Tanvir Hossain, Caroline Chasara, Trevor Khaba, Bongiwe Mahlobo, Nicole Reddy, Kavidha Reddy, Thandekile Ngubane, Johan Pansegrouw, Crystal A. Mendoza, Paradise Madlala, Thumbi Ndung’u, Tokameh Mahmoudi, Zaza M. Ndhlovu

**Affiliations:** 1 Africa Health Research Institute (AHRI), Nelson R. Mandela School of Medicine, University of KwaZulu-Natal, Umbilo, Durban, South Africa.; 2 Department of Biochemistry, Erasmus University Medical Center, Rotterdam, The Netherlands.; 3 HIV Pathogenesis Programme, Doris Duke Medical Research Institute, Nelson R. Mandela School of Medicine, University of KwaZulu-Natal, Umbilo, Durban, South Africa.; 4 Department of Surgery, Prince Mshiyeni Memorial Hospital, Umlazi, Durban, South Africa.; 5 Ragon Institute of MGH, MIT, and Harvard, Cambridge, MA, USA.; 6 University College London, London, United Kingdom; 7 Department of Urology, Erasmus University Medical Center, Rotterdam, The Netherlands.; 8 Department of Pathology, Erasmus University Medical Center, Rotterdam, The Netherlands.

## Abstract

Despite durable viral suppression, cellular sources of human immunodeficiency virus type 1 (HIV-1) persistence in human lymphoid tissues remain uncharacterized, with germinal center (GC) macrophages representing an understudied, potentially noteworthy reservoir population. Using integrated spatial, phenotypic, and molecular profiling of 42 excisional lymph nodes (LNs) from 39 individuals living with HIV-1 Clade C, including 25 under suppressive antiretroviral therapy (ART), we reveal that GC macrophages harbor HIV-1 DNA, RNA, and proteins, defining them as a functional HIV reservoir. Super-plex imaging confirms HIV co-localization with GC macrophages, which were predominantly IBA-1^+^CD68^+^and CD206^−^CD163^−^. Among individuals with sustained viral suppression, the presence of proviral DNA (3/6) and inducible multiply spliced HIV-1 RNA (3/3) confirms that LN-resident myeloid cells serve as inducible HIV-1 reservoirs. These reservoirs were markedly enriched in the LNs, with both CD4^+^ and myeloid compartments showing higher inducible activity than their peripheral blood counterparts. While CD4^+^ T cells remain the predominant source of inducible, replication-competent HIV, our detection of a LN myeloid reservoir underscores the imperative to redesign cure approaches to eliminate reservoirs across both CD4^+^ T cell and myeloid lineages.

## Introduction

Comprehensive characterization of HIV reservoirs is essential for the success of curative strategies (1). Lymph node (LN) tissues serve as key sanctuary sites for HIV reservoir persistence during suppressive antiretroviral therapy (ART). Poor ART penetration and suboptimal immune responses pose major challenges to the eradication of the HIV reservoir in lymph nodes (LNs) (2). Although very early ART initiation has been shown to accelerate HIV reservoir decay, low-level HIV replication persists in secondary lymphoid organs (3, 4). We previously showed that T follicular helper (Tfh) cells in germinal centers (GCs) are the dominant HIV reservoirs in LNs of individuals who initiated ART during hyperacute infection, and that poor CXCR5-dependent trafficking of cytotoxic CD8^+^ T cells into GCs limits immune-mediated clearance (5, 6). Here, we asked whether LN-resident macrophages also sustain persistent HIV reservoirs, motivated by evidence that macrophages can harbor productive infection during suppressive ART and reside in anatomical niches, including GCs, where reservoir persistence is concentrated (1, 5, 7–11). The identification of long-lived, embryonically derived macrophages, combined with their inherent resistance to cytotoxic T lymphocyte (CTL)-mediated clearance and permissiveness to HIV, further emphasizes their significance as a stable HIV reservoir (12–22).

Macrophages are highly diverse, with distinct phenotypes that differ in their contributions to HIV reservoirs (11, 20). Their physiological functions and spatial localization influence this variability within their respective microenvironment (23). Classification of macrophages into pro-inflammatory or anti-inflammatory states is complex, as a variety of markers can be used to distinguish these phenotypes, including CD68^+^/CD80^+^/CD86^+^ for pro-inflammatory and CD206^+^/CD163^+^/CD200^+^ for anti-inflammatory (24–28). Common classification strategies to delineate macrophage subtypes include using CD68 as a pan-macrophage marker and CD206 to characterize anti-inflammatory macrophages (12, 24, 25). More recent studies have focused on the use of the Ionized calcium-binding adapter molecule 1 (IBA-1), a key marker of microglia, as an alternative marker of tissue-resident macrophages in rats (29).

While cumulative research has established the existence of replication-competent, long-lived HIV reservoirs in macrophages (11, 30, 31), the precise mechanisms and extent of their involvement in sustaining viral persistence remain contentious. These limitations stem from both the scarcity of intact tissues and the challenges of using high-resolution spatial tools to phenotype and spatially resolve macrophages that maintain HIV reservoirs. To overcome these limitations, this study utilized a total of 61 excisional LN tissue samples from 58 participants (with n=3 repeat participants, each donating a second LN), including 39 people living with HIV (PLWH) and 19 demographically matched people living without HIV (PLWoH). Using a combination of integrated imaging and molecular assays, we phenotypically characterized LN macrophages according to their reservoir-relevant spatial niches in the GC and probed their potential to harbor previously undescribed proviral and inducible reservoirs.

Here, we report that persistent HIV infection is associated with a marked expansion of macrophages within LNs, and that IBA-1^+^CD68^+^CD206^−^CD163^−^ macrophages located in GCs harbor proviral DNA, viral RNA, and HIV Gag proteins, establishing them as bona fide LN HIV reservoirs. We further show that proviral HIV DNA is detectable in myeloid cells in most individuals and that the inducible HIV reservoir is substantially enriched in LNs, across both CD4^+^ T cells and myeloid cells, as compared to peripheral blood. These findings highlight the critical role of GC macrophages as a reservoir for HIV in LNs during suppressive ART, indicating a need to include this subset in future HIV eradication research aimed at achieving a cure.

## Results

### Participant demographics

We recruited 58 participants from the Females Rising through Education Support and Health (FRESH) and HIV Pathogenesis Programme (HPP) Acute study cohorts. Participants were stratified based on Fiebig stage and treatment status: PLWoH (n=19), individuals treated during Fiebig stage I-V (i.e., acute-treated PLWH; [n=6]), and individuals treated after Fiebig stage V (i.e., chronic-treated PLWH; [n=27]). Two PLWH were untreated, and four PLWH had an unknown treatment status at the time of sample collection. Notably, three chronic-treated participants donated a repeat sample at a different timepoint, amounting to a total of 61 excisional LNs investigated in this study.

The demographic and clinical characteristics of the participants, including sex, age, LN site, plasma viral load (pVL), CD4^+^ T cell count, ART timing, and ART duration before LN excision, are presented in Table 1 and expanded in **Supplementary Table 1**. The pVL was detectable in 1/6 (16.7%) acute-treated PLWH. From the chronic-treated PLWH, the pVL was detectable in 9/24 PLWH (37.5%) and in the return participants (n=3), pVL was detectable at both timepoints (n=1) or one timepoint (n=2) **(Supplementary Table 1)**.

### Impact of HIV infection on the phenotypes, location, and frequencies of macrophages in human LN tissues

**Figure 1.**
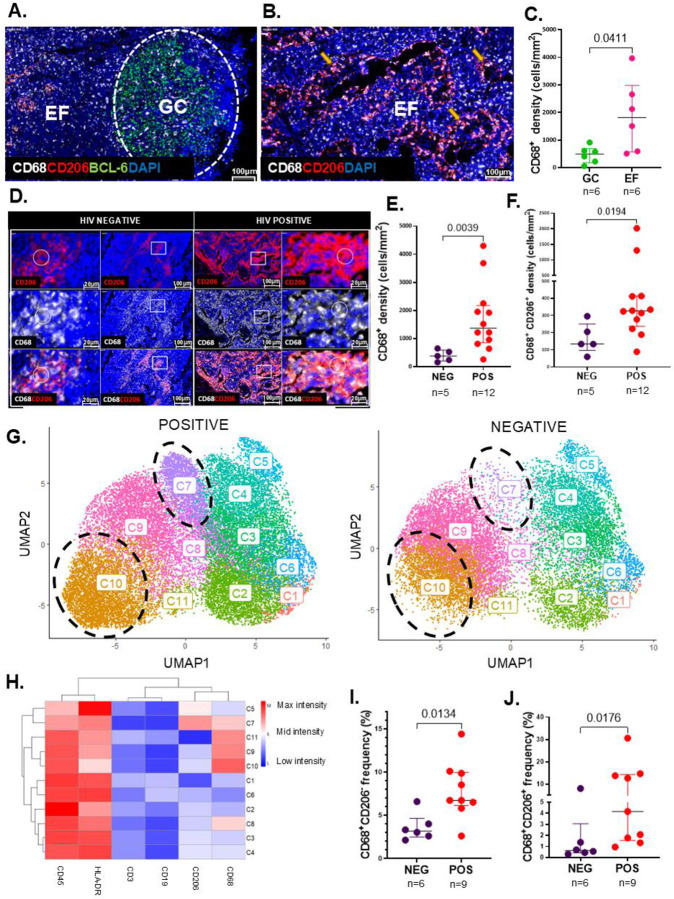
Impact of HIV infection on the phenotypes, location and frequencies of macrophages in human LN tissues. **(A)** Representative multicolor IF image of the localization of CD68^+^ (white) and CD206^+^ (red) macrophages relative to the LN GC (white dashed oval) indicated by BCL-6 (green) positivity or the extrafollicular (EF) region (outside the GC). **(B)** Representative multicolor IF image of CD68^+^CD206^+^ macrophages along LN lymphatic and blood vessels (yellow arrows). **(C)** Aggregate data from quantitative image analysis by TissueQuest (TissueGnostics, Vienna) indicating the density of CD68^+^ macrophages (cells/mm^2^) which localized inside the GC (green) and the EF region (pink) from LN tissues of n=6 different donors, p=0.0411. **(D)** Multicolor IF images of the individual channels of CD206^+^ macrophages (red), CD68^+^ macrophages (white), and the composite image of CD68^+^CD206^+^ macrophages in LN tissue sections from a representative PLWoH and a PLWH. The white rectangles from the inner panels indicate regions of interest which are further magnified on the outer panels. The white circles on the outer panels indicate the double positive CD68^+^CD206^+^ macrophages**. (E-F)** Quantitative image analysis by TissueQuest (TissueGnostics, Vienna) between n=5 PLWoH (purple) and n=12 PLWH (red) of the frequency (cells/mm^2^) of: CD68^+^ macrophages, p=0.0039 **(E)** and CD68^+^CD206^+^ macrophages, p=0.0194 **(F)**. **(G)** Multiparametric global Uniform Manifold Approximation and Projection (UMAP) visualization of LNMCs from 9 PLWH and 6 PLWoH. The Flow Self-Organizing Map (FlowSOM) was overlaid on the UMAP which identified 11 clusters of LNMCs and are labelled on the UMAP. **(H)** The unsupervised hierarchical clustering heatmap summarizes the mean fluorescence intensities of each loaded parameter (CD3, CD19, CD45, HLA-DR, CD68 and CD206), whereby warm colors (dark red) indicate high expression and cold colors (dark blue) indicate low expression. **(I-J)** Frequency of parent (%) comparison between n=6 PLWoH (purple) and n=9 PLWH (red) of CD68^+^CD206^−^ macrophages, p=0.0134 **(I)** and CD68^+^CD206^+^ macrophage frequency, p=0.0176 **(J)** within LNMCs. Images were acquired at 40x and all cell nuclei were detected by DAPI (blue). Scale bars= 100μm and 20μm. Comparisons made using the Mann Whitney tests, whereby horizontal bars denote the median, error bars represent the interquartile range (IQR) and each circle represents an individual donor. Levels of significance are as follows: *: p<0.05; **: p<0.01 and ***: p<0.001.

LN GCs are a key anatomical site of HIV reservoir persistence (5, 32). While macrophages are ubiquitous in this microanatomical site, the precise phenotypic characteristics and spatial distribution of macrophages susceptible to HIV infection have not been comprehensively characterized in human LNs (33). We first assessed how HIV infection reshapes macrophage phenotypes and spatial distribution within LNs. Using multicolor immunofluorescence, we quantified pro-inflammatory (CD68^+^CD206^−^) and anti-inflammatory (CD68^+^CD206^+^) macrophages and used BCL-6 to define active GCs (12, 24, 25). Representative images revealed a pronounced spatial segregation: CD68^+^CD206^−^ macrophages were concentrated within follicular regions, whereas CD68^+^CD206^+^ macrophages localized predominantly to extrafollicular, perivascular areas, close to lymph and blood vessels (Fig. 1A–B **and Supplementary Fig. 1**). Quantitative image analysis using TissueQuest **(Supplementary Fig. 2)** confirmed that the density (cells/mm^2^) of CD68^+^ macrophages was significantly higher within the EF regions than in GCs (p=0.0411) (Fig. 1C).

Given the immune-modulatory effects of HIV (34, 35), we examined the impact of HIV on macrophage polarization and frequency in LN tissues using multicolor IF microscopy combined with TissueQuest quantitative image analysis. We stained LN sections for CD68, CD206, and DAPI from six individuals (n=3 PLWoH and n=3 PLWH) as shown by representative images in Fig. 1D, **and Supplementary Fig. 3A**. We calculated macrophage density (cells/mm^2^) in 5 PLWoH and 12 PLWH (see **Supplementary Table 1** for donor information) using TissueQuest image analysis software. Aggregate data for 12 PLWH show greater densities of CD68 and CD206 single and double positive populations compared to 5 PLWoH (CD68^+^ p=0.0039, and CD68^+^CD206^+^ p=0.0194) (Fig. 1E–F).

To substantiate the imaging data, we isolated lymph node mononuclear cells (LNMCs) from nine PLWH and six PLWoH, selected based on sample availability, and performed flow cytometry. The gating strategy is illustrated in **(Supplementary Fig. 3B)**. Availability of more markers allowed for a more detailed granular characterization of pro-inflammatory (CD3^−^CD19^−^CD45^+^HLA-DR^+^CD68^+^CD206^−^) and anti-inflammatory (CD3^−^CD19^−^CD45^+^HLA-DR^+^CD68^+^CD206^+^) macrophage subsets. The UMAP analysis categorized the LNMC populations into 11 cell clusters (Fig. 1G–H). CD45^+^HLA-DR^+^CD3^−^CD19^−^CD68^+^CD206^−^ pro-inflammatory macrophages were detected in cell cluster C10. CD45^+^HLA-DR^+^CD3^−^CD19^−^CD68^+^CD206^+^ anti-inflammatory macrophages were detected in cell cluster C7 (Fig. 1G). Clear distinctions in cell clustering were observed: C7 cells, with a CD68^+^CD206^+^ phenotype, and C10 cells, with a CD68^+^CD206^−^ phenotype, both demonstrated higher densities in PLWH than in PLWoH (Fig. 1G). Quantitative analysis confirmed the elevated levels of these two populations in PLWH: CD68^+^CD206^−^ (C10) (p=0.0134) (Fig. 1I) and CD68^+^CD206^+^ (C7) (p=0.0176) (Fig. 1J **and Supplementary Fig. 3C**), corroborating the imaging data. Collectively, these data show that HIV infection markedly expands pro-inflammatory macrophages in human LNs, a shift that persists despite early ART.

### HIV-1 persists in IBA-1^+^CD68^+^ LN-resident germinal center macrophages despite early ART initiation

**Figure 2.**
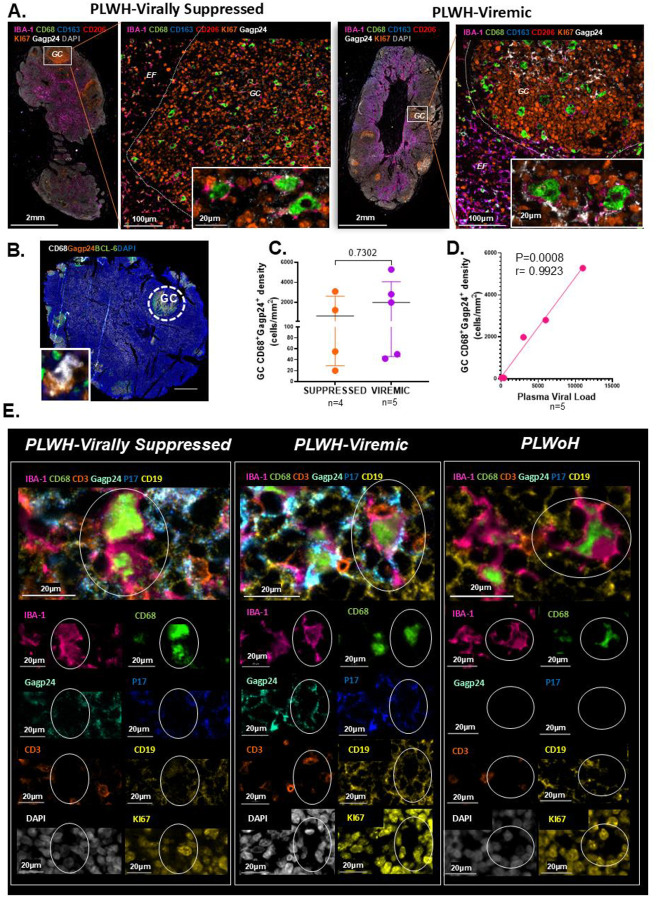
HIV-1 persists in bona fide IBA-1^+^CD68^+^ lymph node germinal center macrophages despite ART. **(A)** Representative Lunaphore COMET whole LN tissue sections from a virally suppressed (left) and viremic (right) PLWH of macrophage subsets defined by IBA-1 (pink), CD68 (green), CD163 (blue), CD206 (red) in relation to the germinal center (GC) denoted by KI67 (orange) positivity or extrafollicular (EF) regions and HIV Gagp24 (white). For each whole LN tissue section, a GC is magnified, with further magnification (bottom right insets) showing IBA-1+CD68+ macrophage co-localization with HIV Gagp24 in GCs. **(B)** Representative multicolor IF image of a whole LN tissue section of CD68^+^ (white) macrophages, HIV Gagp24 antigen (brown) and BCL-6 (green) to define GCs. Co-localization of a CD68^+^ macrophage with HIV Gagp24 antigens within the GC is further magnified (bottom-left inset). **(C)** Aggregate quantitative image analysis data by TissueQuest (TissueGnostics, Vienna) comparing the density of GC CD68^+^Gagp24^+^ macrophages (cells/mm^2^) in n=4 virally suppressed (orange) and n=5 viremic (purple) PLWH, p=0.7302. **(D)** Correlation between the density of GC CD68^+^Gagp24^+^ macrophages (cells/mm^2^) and plasma viral load (VL) in n=5 PLWH (pink), p=0.0008, r=0.9923 by Spearman’s ranked correlation analysis. **(E)** Representative Lunaphore COMET merged and individual channel images from a virally suppressed (left) PLWH and viremic PLWH (middle) of bona fide GC macrophages denoted by positivity for IBA-1 (pink), CD68 (green) and negativity for CD3 (orange) and CD19 (yellow), co-localizing with HIV Gagp24 (mint) and Gagp17 (dark blue). A control representative image from a PLWoH is shown (right). Individual channels for KI67 (yellow) to denote GC localization and nuclei counterstained with DAPI (grey) are shown. Images were acquired at 20x or 40x magnification and cell nuclei were detected by DAPI (blue or grey). Scale bars equal to 2mm, 20 μm, 100μm and 200μm. Comparisons made using the Mann Whitney tests, whereby horizontal bars denote the median, error bars represent the interquartile range (IQR), and each circle represents an individual donor. Levels of significance are as follows: *: p<0.05; **: p<0.01 and ***: p<0.001.

We next asked whether the expanded macrophage populations harbor HIV under suppressive ART. LNs from six PLWH (n=3 virally suppressed; n=3 viremic) and PLWoH as controls (n=3) were analyzed, selected for tissue quality, GC presence, and treatment status. Macrophage subsets were further defined using IBA-1, a marker of tissue-resident macrophages (29), and CD163, which identifies immunoregulatory macrophages (36). HIV Gagp24 detected infected cells, and Ki67 marked active GCs. The Lunaphore COMET technology was used for these analyses, as it allows for high-plex multicolor immunofluorescence panel design (37). Representative images reveal the dominant GC macrophage phenotype as IBA-1^+^CD68^+^CD206^−^CD163^−^ with detectable Gagp24 co-localization in both virally suppressed and viremic PLWH (Fig. 2A). As expected, control LN tissue showed no detectable Gagp24 staining (**Supplementary Fig. 4A**). We observed rare occurrences of IBA-1^+^CD68^+^CD206^−^CD163^+^ macrophages in LN GCs, which were mostly located in the dark zone of PLWoH, viremic PLWH (**Supplementary Fig. 4B-C**) and virally suppressed PLWH (**Supplementary Fig. 5A**). HALO FISH IF spatial analyses supported the rarity of this population in LN GCs, which had no detectable Gagp24^+^ density (**Supplementary Fig. 5B-D**). In contrast, Gagp24 positivity was detectable in GC IBA-1^+^CD68^+^CD206^−^CD163^−^ macrophages, which constituted approximately 2% of the infected cell proportion as compared to GC CD3^+^CD4^+^Gagp24^+^ T cells (**Supplementary Fig. 5E-G**).

Using low-plex, four-color immunofluorescence microscopy, we quantified HIV-infected macrophages across a larger sample size, measured GC Gagp24^+^ macrophage density, stratifying participants by plasma viral suppression at LN excision (**Supplementary Table 1**). LN tissues from three PLWoH showed no detectable Gagp24 signal, serving as a Gagp24 staining control condition **(Supplementary Fig. 6A)**. Active GC and extrafollicular regions were delineated by BCL-6 staining, macrophages were marked with CD68, and HIV protein expression was visualized using Gagp24. CD68^+^ macrophages staining positive for HIV Gagp24 protein were once again readily detectable, almost exclusively in LN GCs, as shown in the representative images (Fig. 2B and **Supplementary Fig. 6B**).

Building on evidence that HIV persists predominantly in LN GCs even when ART is initiated in hyperacute infection and plasma viremia is suppressed (5), we assessed the macrophage reservoir size based on the peripheral virus-suppression status. Quantitative analysis by TissueQuest revealed no significant difference between individuals with complete plasma viral suppression and those who were viremic (p=0.7302) (Fig. 2C). In viremic individuals, notably, the density of HIV Gagp24^+^ macrophages correlated with pVL (p=0.0008; r=0.9923) (Fig. 2D). Notably, when we expanded our staining to include HIV Gagp17 as a marker of HIV infected cells, we saw a similar trend as with HIV Gagp24 expression in GC macrophages absent of CD3 and CD19 expression (Fig. 2E and **Supplementary Fig. 7**) in LNs from PLWH (n=6).

Together, these robust phenotypic and spatial characterizations define GC macrophages as true tissue-resident macrophages, with a majority exhibiting an IBA-1^+^CD68^+^CD206^−^ phenotype with evidence of dual HIV antigen co-expression. Their potential role towards HIV reservoir contribution is exacerbated during late ART onset, which highlights the importance of early-ART initiation.

### Detection of HIV subtype C proviral DNA and HIV-1 transcriptional activity within LN macrophages

**Figure 3.**
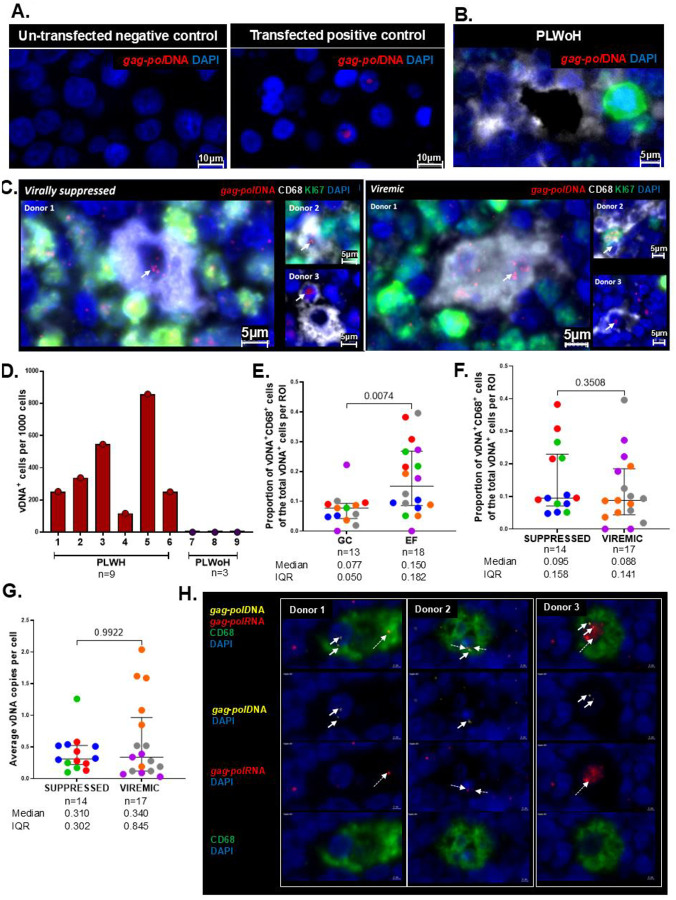
Detection of HIV subtype C proviral DNA within lymph node macrophages. **(A)** FISH DNAscope of *gag-pol* HIV DNA (red) in negative and positive controls using un-transfected and HIV-transfected cell lines respectively. **(B)** Representative FISH DNAscope image from a PLWoH of *gag-pol* HIV DNA (red), CD68 (white), KI67 (green) and nuclei counterstained in DAPI (blue). **(C)** Representative images of FISH DNAscope coupled with multicolor IF of *gag-pol* HIV subtype C DNA (red) (white arrows), Ki67 (green), and CD68 (white) detection within LN tissues from n=3 virally suppressed and n=3 viremic PLWH. **(D)** Aggregate data of the frequency of *gag-pol*DNA^+^ (vDNA^+^) cells per 1000 cells between n=6 PLWH (red) and n=3 PLWoH (purple) using HALO image analysis. **(E)** The proportion of vDNA^+^CD68^+^ cells out of the total vDNA^+^ cells per ROI between n=13 GC and n=18 EF regions from n=3 suppressed and n=3 viremic PLWH, p=0.0074. **(F)** The proportion of vDNA^+^CD68^+^ cells out of the total vDNA^+^ cells per ROI between n=14 regions from n=3 suppressed and n=17 regions from n=3 viremic PLWH, p=0.3508. **(G)** Average vDNA copies per cell between n=14 regions from n=3 suppressed and n=17 regions from n=3 viremic PLWH, p=0.9922. **(H)** Representative merged and individual channel images of multiplexed DNAscope and RNAscope staining from n=3 virally suppressed PLWH of *gag-pol* HIV subtype C DNA (yellow), *gag-pol* HIV-1 RNA (red), CD68 (green) and nuclei counterstained in DAPI (blue) showing co-expression of HIV vDNA (solid arrows) and vRNA (dashed arrows) in CD68^+^ macrophages from LN tissues. Images were acquired at 40x and all cell nuclei were detected by DAPI (blue). Scale bars= 5μm and 10μm. Comparisons made using the Mann Whitney tests, whereby horizontal bars denote the median, error bars represent the interquartile range (IQR). Each color represents an individual donor and each circle represents an individual ROI. Levels of significance are as follows: *: p<0.05; **: p<0.01 and ***: p<0.001.

Next, we used *in situ* DNAscope and RNAscope FISH to detect latent and transcriptionally active reservoirs, respectively. We employed an HIV *gag-pol* sense probe optimized for HIV Clade C, which had a 92.8% alignment with sequences from our study cohort **(Supplementary Fig. 8A)** and validated the specificity of the DNA probe in an *in vitro* cell culture model (Fig. 3A). For the DNAscope studies, we utilized six LNs from PLWH (n=3 virally suppressed; n=3 viremic) and LNs from PLWoH as controls (n=3). As expected, no vDNA was detected in the LNs from PLWoH (Fig. 3B and **Supplementary Fig. 8B**). Representative images and aggregate data show that all six LNs from PLWH contained varying levels of detectable subtype C *gag-pol* vDNA (Fig. 3C–D). DNA punctate signals were predominantly localized within the nuclei of CD68^+^ macrophages from PLWH (Fig. 3C). Next, we mapped the spatial distribution of macrophages harboring proviral reservoirs using a HALO-customized FISH-IF module. Unlike our protein results, the vDNA signal was observed in both follicular and EF zones, with the median fraction of vDNA^+^ macrophages among the total vDNA^+^ cells measuring 0.077 in GCs and 0.150 in EF regions (p=0.0074) (Fig. 3E). We then assessed the impact of plasma suppression status on the proportion of vDNA^+^ macrophages of the total vDNA^+^ cells per zone and found no significant difference (p=0.3508) (Fig 3F). Additionally, we observed multiple punctate vDNA signals within the nuclei, which are thought to represent different HIV integration sites across the genome (38). Given that viremic PLWH are more likely to experience viral reactivation, we compared vDNA copies per cell between the viremic and virally suppressed groups. We observed no significant difference across groups (p=0.9922) (Fig 3G).

Next, we multiplexed DNAscope and RNAscope FISH on the same LN sections to determine whether vDNA^+^ macrophages are transcriptionally active and capable of driving viral rebound (39). This analysis was done on LNs obtained from n=3 virally suppressed PLWH, and further applied immunofluorescence staining with CD68, a classical macrophage marker. As exemplified by merged and individual channel images, we identified evidence suggestive of potential HIV-1 reservoir transcriptional activity in all samples from suppressed individuals tested, as evidenced by positive *gag-pol* DNA and *gag-pol* RNA signals (39, 40) within CD68^+^ macrophages (Fig. 3H).

### HIV proviral DNA quantification and comparison between LN myeloid and CD4^+^ T cells

**Figure 4.**
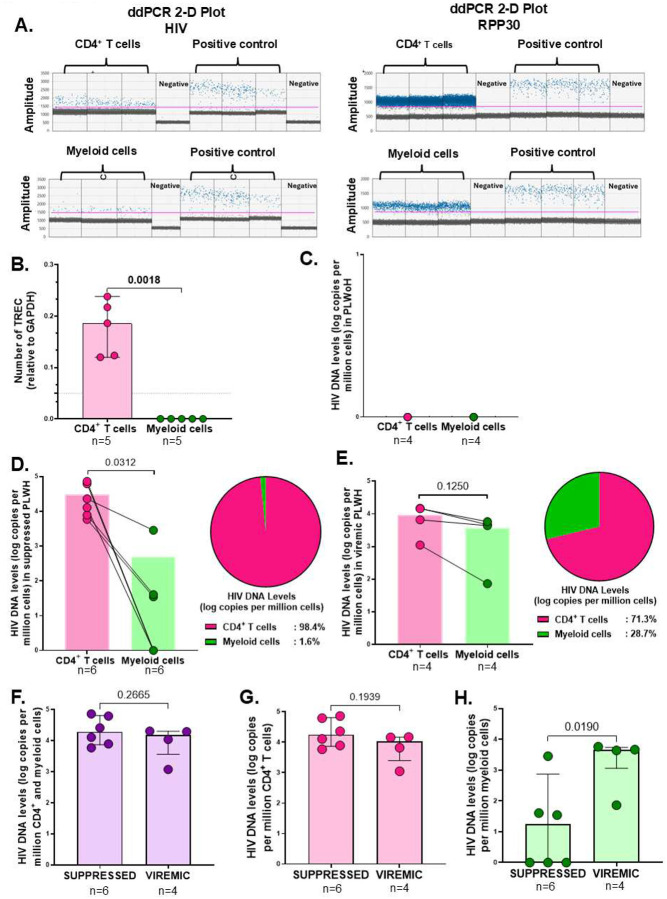
HIV proviral DNA quantification and comparison between lymph node myeloid and CD4^+^ T cells. **(A)** Representative 1-D ddPCR plot from a viremic PLWH showing droplets from CD4^+^ T and myeloid cell samples, with each droplet from a sample plotted on a graph of fluorescence intensity versus droplet number. Blue dots denote positively amplified droplets while grey dots are negative. A manual threshold indicated by the pink line was set to distinguish the positive and negative droplet clouds. DNA from the 8E5/LAV cell line was used as a positive control. **(B-C)** ddPCR controls: Number of TREC relative to GAPDH, p=0.0018 by Student’s paired T test. **(B)** and HIV DNA levels (log copies per million cells) in n=4 PLWoH between CD4^+^ T cells (pink) and myeloid cells (green) **(C)**. **(D)** Aggregate data comparing HIV DNA levels (log copies per million cells) in n=6 virally suppressed PLWH between CD4^+^ T cells (pink) and myeloid cells (green), p=0.0312 by Wilcoxon rank sum test. The pie chart (right) depicts the proportion of HIV DNA levels in the CD4^+^ T cells (pink) compared to myeloid cells (green) in virally suppressed PLWH. **(E)** Aggregate data comparing HIV DNA levels (log copies per million cells) in n=4 viremic PLWH between CD4^+^ T cells (pink) and myeloid cells (green), p=0.1250 by Wilcoxon rank sum test. The pie chart (right) depicts the proportion of HIV DNA levels in the CD4^+^ T cells (pink) compared to myeloid cells (green) in viremic PLWH. **(F-H)** Aggregate data comparing HIV DNA levels (log copies per million cells) in n=6 virally suppressed PLWH and n=4 viremic PLWH, in pooled CD4^+^ T cells and myeloid cells, p=0.2665 by Students unpaired t test **(F)**, in isolated CD4^+^ T cells, p=0.1939 by Students unpaired t test **(G)** and in isolated myeloid cells, p=0.0190 by Mann Whitney test **(H). (B; H)** Bars denote the median and error bars represent the interquartile range (IQR). **(F; G)** Bars indicate the mean and error bars denote the standard deviation. Levels of significance are as follows: *: p<0.05; **: p<0.01 and ***: p<0.001.

To further substantiate the role of macrophages in maintaining the latent tissue reservoir, we measured total proviral DNA levels in myeloid cells and CD4^+^ T cells derived from LNs using digital droplet polymerase chain reaction (ddPCR), a method typically used to quantify the total proviral reservoir (41). CD4^+^ T cells and paired myeloid cell subsets were FACS-sorted from LNMCs, as outlined in the gating strategy **(Supplementary Fig. 9A)**. We sorted the total myeloid population rather than macrophage subsets due to the insufficient yield of purified macrophage populations from the tissues required for the ddPCR assay (12). Ten samples from PLWH (n=6 suppressed; n=4 viremic) were selected based on peripheral suppression status and sample availability and used for these studies. The ddPCR results showed HIV proviral DNA levels (log copies/ million cells) within both the CD4^+^ T cell and myeloid cell compartments (41) as indicated by the representative ddPCR 1-D plot from a viremic individual (Fig. 4A). Assay validatory checks by TREC PCR 2-D plots **(Supplementary Fig. 9B-C)** and quantification revealed no CD4^+^ T cell contamination within the sorted myeloid cells (Fig. 4B). Assay control testing from samples from PLWoH (n=4) yielded no detectable HIV DNA as expected (Fig. 4C). We detected proviral HIV DNA in the CD4^+^ T cell population in all samples from PLWH (n=10). In the myeloid compartment, proviral HIV DNA was detected in 7 of 10 PLWH (3/6 virally suppressed and 4/4 viremic). In virally suppressed PLWH (pVL<20 copies/mL) the proportion of HIV DNA levels was higher in the CD4^+^ T cell population (98.4%) compared to the myeloid cell population (1.6%) (p=0.0312) (Fig. 4D). In viremic PLWH (pVL>250), we observed a similar trend of higher HIV DNA levels in the CD4^+^ T cell population (71.3%) compared to the myeloid cell population (28.7%) (p=0.1250) (Fig. 4E). Notably, the proportion of proviral DNA from the myeloid compartment was higher in viremic PLWH compared to suppressed PLWH (Fig. 4D–E). HIV proviral DNA levels were similar in viremic compared to suppressed PLWH in the pooled CD4^+^ T cell and myeloid cell population (p=0.2665) (Fig. 4F) and within the CD4^+^ T cell population (p=0.1939) (Fig. 4G). However, in the myeloid cell population, HIV DNA levels were higher in viremic compared to virally suppressed PLWH (p=0.0190) (Fig. 4H). Our findings reveal that the myeloid compartment harbors about 29% of the total proviral DNA in LNs of viremic individuals, and only 1.6% of the total proviral DNA in LNs of virally suppressed, suggesting a marked reduction in proviral burden within myeloid cells during suppressive ART.

### Quantification of functional inducible reservoir properties of myeloid and CD4^+^ T cells in paired LMNCs and PBMCs

Finally, to determine whether LN myeloid cells harbor inducible, functional HIV reservoirs compared to CD4^+^ T cells in the same compartment and in the blood, we performed the novel SQuHIVLa assay (Specific Quantification of inducible HIV-1 reservoir by RT-LAMP) (42). The SQuHIVLa assay is a scalable, high-throughput HIV-1 reservoir quantification tool with high sensitivity and specificity, which detects and quantifies the expression of *tat/rev* HIV-1 multiply spliced RNA (msRNA) in activated cells (42). Defective proviruses often carry 3’ mutations or deletions in the *tat* and *rev* genes, rendering them incapable of generating msRNA, and translation of Tat is essential for productive infection (43, 44). Thus, quantification of reservoir cells able to produce msRNA filters out the vast majority of cells harboring defective proviruses (45). This subpopulation of msRNA expressing reservoir cells have also been shown to correlate with viral rebound during ART interruption (46).

**Figure 5.**
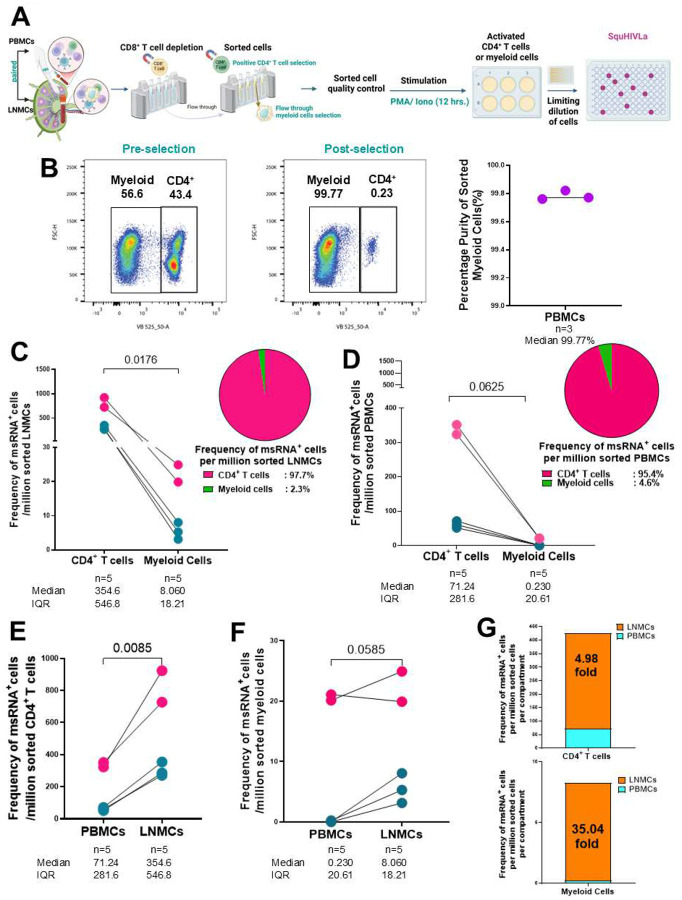
Specific quantification of inducible HIV-1 reservoir by RT-LAMP (SQuHIVLa) through multiply spliced HIV-1 RNA (msRNA) detection in sorted LNMCs and paired PBMCs. **(A)** Schematic of paired sampling of PBMCs and LNMCs which underwent CD8^+^ T cell depletion and cell sorting into CD4^+^ T cell and myeloid cell compartments and tested for quality control. Sorted cells were stimulated with PMA/ Iono for 12 hours and the activated cells underwent a limiting dilution, followed by the SQuHIVLa assay. **(B)** Flow cytometry gating strategy. **(C)**The inducible HIV-1 reservoir was quantified as the frequency of msRNA^+^ cells /million sorted LNMCs obtained from n=3 virally suppressed PLWH (blue) and n=2 viremic PLWH (pink) and compared using the Wilcoxon rank sum test between sorted CD4^+^ T cells and myeloid cells p=0.0176. The pie chart (right) depicts the proportion of msRNA^+^ cells /million sorted LNMCs in the CD4^+^ T cells (pink) compared to myeloid cells (green) from PLWH. **(D)** Aggregate data **of the** frequency of msRNA^+^ cells /million sorted and paired PBMCs compared using the Wilcoxon rank sum test p=0.0625. The pie chart (right) depicts the proportion of msRNA^+^ cells /million sorted and paired PBMCs in the CD4^+^ T cells (pink) compared to myeloid cells (green) from PLWH. **(E-F)** The Student’s paired t test was used to compare the frequency of msRNA^+^ cells /million sorted cells between paired PBMCs and LNMCs compartments for the CD4^+^ T cells (p=0.0085) and myeloid cells (p=0.0585). **(G)** Bar plots showing the median fold changes between LNMC (orange) and PBMC (blue) compartments of the frequency of msRNA^+^ cells /million sorted CD4^+^ T cells and myeloid cells. Each dot represents an individual LNMC donor. Levels of significance are as follows: *: p<0.05; **: p<0.01 and ***: p<0.001. Panel A created in BioRender. Mendoza-Lopez, C. and Moodley, M. (2025) https://BioRender.com/0ijyrdh

Here, we utilized magnetic cell separation to isolate, sort and stimulate paired LNMCs and PBMCs from five PLWH (n=3 virally suppressed; and n=2 viremic) for the SQuHIVLa assay (Fig. 5A and **Supplementary Fig. 9D**). Following CD8^+^ T cell depletion, cell sorting validation checks revealed no significant CD4^+^ T cell contamination in the sorted myeloid cells, as denoted by the flow plots and aggregate data from three participants, yielding a median of 99.77% purity of myeloid cells (Fig. 5B, **right**). msRNA^+^ cells were readily detectable in all samples from PLWH (n=5) in both the myeloid and the CD4^+^ T cell populations (Fig. 5C). The frequency of inducible msRNA^+^ cells (per million sorted cells) was lower in the myeloid cell compartment (median= 8.060) as compared to the CD4^+^ T cell compartment (median= 354.6) in LNMCs (p=0.0176) (Fig. 5C). In paired PBMCs, msRNA^+^ cells were readily detectable in all PLWH in the CD4^+^ T cell population but only detected in 60% of PLWH in the myeloid population (n=3/5 total; n=2/2 viremic; n=1/3 virally suppressed) (Fig. 5D). In paired PBMCs, the frequency of inducible msRNA^+^ cells (per million sorted cells) was lower in the myeloid cell compartment (median= 0.23) compared to the CD4^+^ T cell compartment (median= 71.24; p=0.0625) (Fig. 5D). The frequency of inducible msRNA^+^ cells per million sorted CD4^+^ cells was higher in LNMCs (median=354.6) compared to paired PBMCs (median=71.24; p=0.0085) (Fig. 5E). Furthermore, the frequency of inducible msRNA^+^ cells per million sorted myeloid cells was higher in LNMCs (median=8.060) compared to paired PBMCs (median=0.230; p=0.0585) (Fig. 5F). Finally, we found that the median frequency of inducible msRNA^+^ cells per million sorted CD4^+^ T cells and myeloid cells were higher in the LNMCs as compared to matched PBMCs by 4.98-fold and 35.04-fold respectively (Fig. 5G). Overall, the results indicate that inducible HIV reservoirs are significantly enriched in CD4^+^ and myeloid cells within the LN microenvironment relative to the peripheral reservoir, underscoring the central role of lymphoid tissues in sustaining HIV persistence.

## Discussion

The eradication of HIV remains challenging due to the virus’s ability to establish and persist in reservoir sites, particularly within lymphoid tissues (1, 5). Understanding the role of understudied cell types, such as macrophages, in maintaining HIV reservoirs in tissues is essential for achieving complete HIV eradication. In this study, we characterized the phenotype, localization, and reservoir nature of macrophages in the LN tissues of individuals with suppressed HIV clade C infection (1, 47). Our findings demonstrate that IBA-1^+^CD68^+^ CD206^−^ GC LN macrophages harbor proviral, transcriptionally active, and inducible reservoirs, highlighting their critical role in sustaining tissue-based viral reservoirs during ART. Importantly, we reveal significant contributions of macrophages to the LN proviral and active reservoirs through robust *in situ* imaging and quantitative analysis, ddPCR, and SQuHIVLa assay.

Previous studies show that CD11c^+^CD68^+^ cells represent about 7% of total cells in the GC (48), supporting the notion that they may serve as an important tissue viral reservoir capable of reactivation upon treatment interruption. To further delineate the macrophage populations contributing to HIV persistence, we used an expanded phenotypic marker panel incorporating CD68, CD206 and CD163 and IBA-1 to characterize macrophages within GCs. This approach uncovered a unique macrophage phenotype (IBA-1^+^CD68^+^CD206^−^CD163^−^) that localized to LN GCs and contained detectable HIV proteins, highlighting a novel pro-inflammatory macrophage subset with the potential to sustain infection within this immune-privileged microenvironment. We also noted a rare GC CD163^+^ macrophage population which was largely HIV Gagp24 negative and spatially restricted to the dark zone. In line with this finding, previous reports show that CD163 expression is suppressed within CD4^+^ T cell–rich niches (49) such as the GC light zone where HIV largely persists (5, 50), potentially reflecting negative regulation by IFN-γ (51) and non-canonical complement enrichment (23). Although CD163^+^ macrophages appear rare in LN GCs, they may play a more prominent role in other HIV infected tissues, such as the brain (52) and spleen (53). Notably, IBA-1 provided clearer delineation of macrophage boundaries and markedly improved their discrimination from adjacent T cells (29). Moreover, viral proteins visualized within IBA-1^+^ macrophage membrane boundaries improved the sensitivity of quantifying the number of infected macrophages within GCs.

The notion of compartmental differences in macrophage phenotypes is consistent with the recent report by Liu et al., where they spatially resolved the whole transcriptomic profile of CD68^+^ macrophages in reactive lymphoid tissues and discovered distinct functional features of macrophages based on their spatial localization (23). Follicular macrophages upregulated genes involved in cell proliferation and metabolism, whereas EF macrophages showed gene enrichment for IFN-γ-associated and TNF-α/NF-κB pathways (23). Notably, higher IFN-γ expression and prolonged effector-target cell contact time impair efficient CTL-mediated killing of macrophages (16), offering a plausible mechanism favoring the persistence of EF macrophage reservoirs.

Furthermore, in contrast to viral protein bearing macrophages, which were tightly restricted to GCs, the hotspot for HIV persistence due to reduced CTL infiltration, our spatial analyses revealed that vDNA^+^ macrophages are dispersed across both follicular and extrafollicular compartments. This distribution suggests that latent HIV infection in macrophages is not confined to immune-privileged niches and may persist even in regions where CTL-mediated surveillance is typically more effective. These findings broaden the current paradigm of tissue-based HIV persistence and imply that macrophage reservoirs may be more anatomically and immunologically widespread, with important implications for reservoir-targeting strategies.

In addition to mapping the spatial architecture of macrophage reservoirs within human lymph nodes, we quantified their relative contribution to the total proviral burden. Quantitative ddPCR analysis revealed that myeloid cells account for approximately 1.6% of the proviral HIV reservoir in LNs of virally suppressed individuals and approximately 28.7% in viremic PLWH when compared to CD4^+^ T cells. This is consistent with a previous study, in urethral macrophages which shows that approximately 1% of macrophages contain integrated proviral DNA and 0.2% to 0.9% of macrophages contain active forms of the reservoir (12). Although the myeloid reservoir is smaller than the CD4^+^ T cell reservoir (14), individuals who are viremic show a significant increase in the ratio of HIV DNA in myeloid cells relative to CD4^+^ T cells. These data collectively suggest that delayed ART initiation shifts the reservoir landscape, increasing the relative role of macrophages in sustaining long-term HIV persistence within tissues.

Conventional functional reservoir assays are limited by large cell inputs, have low sensitivity for rare, infected cells, and disproportionately focus on peripheral blood (54–56). The SquHIVLa assay, as demonstrated here and described by Hossain and colleagues, offers a viable alternative for identifying inducible reservoirs in low-resource settings (42). To our knowledge, this represents the first systematic analysis of inducible reservoir competency in LN sorted CD4^+^ T cells and myeloid cells, benchmarked against matched PBMC populations. This comparative approach offers new insight into compartment-specific differences in reservoir potential. Strikingly, despite being largely undetectable in peripheral blood, myeloid cells exhibited inducible reservoirs in all LNs from fully suppressed individuals, suggesting that they are functional, replication-competent reservoirs. This enrichment of inducible LN myeloid reservoirs underscores both the sensitivity of this approach and the limitations of blood-only reservoir measurements. Incorporating methodologies such as the SQuHIVLa assay to identify mRNA for tat/rev together with gold-standard ddPCR methodologies, may help uncover additional rare inducible reservoirs (42).

This study has several limitations. Most notably, the difficulty in sorting sufficient tissue macrophages for the measurement of total proviral DNA by ddPCR and frequency of inducible msRNA^+^ cells by SQuHIVLa necessitated the use of total myeloid cell population. Although this represents a constraint, its impact is mitigated by the robust purity of the sorted myeloid cells and by the fact that macrophages comprise the majority of this population. Moreover, macrophages remain the only tissue-resident myeloid subset consistently shown to harbor replication-competent virus across human studies and humanized mouse models (57). Furthermore, our study was predominantly conducted among women (4/58 participants were men) from KwaZulu-Natal, South Africa, limiting the generalizability of our findings to other populations, particularly men. Future research should include male participants and broader populations to better understand the interplay between sex, genetic variability, and different HIV subtypes on tissue-based HIV reservoir dynamics. Nonetheless, applying this large dataset to diverse imaging and functional reservoir assays strengthens our conclusion that GC IBA-1^+^ CD68^+^ macrophages are true Subtype C HIV reservoirs.

Overall, this report adds to the mounting evidence that macrophages within lymphoid tissues play a pivotal role as reservoirs for HIV. Particularly, human LN GC IBA-1^+^CD68^+^ macrophages serve as key targets harboring proviral, functional and inducible Clade C HIV-1 reservoirs during full suppression, and significant contributors to viral rebound during viremia. Collectively, these findings underscore the importance of targeting macrophages in future HIV eradication strategies. However, targeting macrophage reservoirs for elimination will require a multifaceted, tissue-specific approach. For instance, the efficacy of reverse-transcriptase (RT) inhibitors and latency-reversing agents (LRAs) differs between CD4^+^ T cells and macrophages (50, 51). Recently, the anti-cancer agent Imatinib was shown to inhibit M-CSF receptor activation, restoring the apoptotic sensitivity of HIV-1-infected macrophages whilst sparing uninfected macrophages, suggesting it as a potentially viable option for specifically targeting macrophage reservoirs (52). This work highlights the importance of characterizing viral reservoir landscapes within other tissues and necessitates the inclusion of the myeloid reservoir in the development of novel therapies for HIV cure.

## Materials and methods

### Sex as a biological variable

This study investigated 58 individuals, of whom 93.10% were women. Due to the limited number of male participants (n=4), this study did not specifically assess sex-based differences during the analysis. Sex is considered a biological variable in the context of HIV (58). In Eastern and Southern Africa, women are three times more likely to acquire HIV than males (58). Therefore, this study predominantly included females, and the overall findings are expected to be interpreted accordingly.

### Study participant details

The participants included in this study (Table 1 and **Supplementary Table 1**) were recruited through two distinct arms. The first arm employed opportunistic recruitment, targeting patients already scheduled for LN excision or endoscopic examination at Prince Mshiyeni Hospital and Durdoc Hospital for diagnostic or therapeutic purposes. The second arm involved recruiting from existing prospective study cohorts within the HIV Pathogenesis Programme (HPP), specifically the Females Rising through Education, Support and Health (FRESH) and ACUTE study cohorts (59). A total of n=61 human LNs were studied from n=58 study participants. Notably, n=3 participants provided a second LN tissue sample at a different time point (**Supplementary Table 1**). The participants were grouped as n=39 PLWH and n=19 PLWoH. The PLWH were further stratified into n=6 acute-treated, and n=27 chronic-treated. Two PLWH were untreated and four PLWH had an unknown treatment status.

### Method details

#### Sample Collection

Excisional LN tissues along with paired blood samples, were collected from these participants exclusively for research purposes as part of the Lymph Node Study (60). Selection criteria were used to select participants and ensure their eligibility for the study. The procedures were carried out safely and in accordance with ethical guidelines by surgical teams at both Prince Mshiyeni and Durdoc Hospitals (60). LN tissue samples were processed and embedded at our laboratories at the Africa Health Research Institute (AHRI), while collected blood samples were forwarded to Neuberg Global for viral load and CD4 count testing.

#### Immunofluorescence (IF) microscopy

Formalin-fixed tissues were first prepared into paraffin-embedded tissue blocks. These FFPE blocks were then sectioned into 4μm slices and affixed onto Surgipath X-tra adhesive pre-cleaned micro slides in preparation for antibody staining. The prepared slides underwent an overnight baking process to soften the paraffin wax and improve tissue adherence onto the glass slide.

Following baking, the slides were deparaffinized in two changes of xylene for 5 minutes each to expose the tissue. Subsequently, the tissue underwent gradual rehydration by immersion in 100% ethanol for 2 minutes, 95% ethanol for 2 minutes, and finally 70% ethanol for 1 minute.

Upon rehydration, the tissue was boiled in 1x EnVision FLEX TRS High pH solution for 20 minutes to expose protein epitopes. Endogenous peroxidases were then blocked using REAL Peroxidase-Blocking Solution for 10 minutes, followed by Bloxallfi Blocking solution for an additional 10 minutes.

Primary antibody was applied, followed by incubation with Opal polymer HRP Ms + Rb secondary antibody for 20 minutes. Detection was done using the Opal polymer range 520, 570, and 690, 10 mins respectively, for each round of staining, followed by counterstaining with a prepared DAPI solution for 5 minutes. For multiplexing with additional antibodies, the tissue underwent boiling in AR6 buffer for 20 minutes to remove the previous antibody complex while preserving the covalently bound TSA fluorescent label, after which the steps were repeated.

Primary antibodies utilized included anti-human BCL-6 (clone PG-B6p, Dako/Agilent Technologies), Ki67 (IR62661, Dako/Agilant Technologies), CD206 (clone 685645, R&D Systems), CD68 (clone KP1, Dako/Agilent Technologies), and p24 (clone Kal-1, Dako/Agilent Technologies Cell Sciences or clone MAP1341, Abnova).

Imaging was conducted using a Zeiss Axio Observer inverted microscope at 40x magnification equipped with a Hamamatsu C13440–20C camera. This was facilitated by TissueFAXS imaging software from TissueGnostics (Vienna, Austria). Quantitative image analysis was performed using TissueQuest (TissueGnostics). Our lab upgraded the Zeiss Axio Observer inverted microscope to the Zeiss Imager Z.2 microscope (TissueGnostics, Vienna, Austria), and the TissueQuest image analysis software (TissueGnostics, Vienna, Austria) to StrataQuest Image Analysis software (TissueGnostics, Vienna, Austria). These upgrades commenced after the initial LN work was completed, and therefore the DNAscope acquisitions were all acquired using the new upgraded system: the Zeiss Imager Z.2 microscope (TissueGnostics, Vienna, Austria).

#### Multicolor flow cytometry (FACS)

Lymph node mononuclear cells (LNMCs) were characterized using multi-parameter flow cytometry analysis. Briefly, cells were stained with LIVE/DEAD Fixable Blue dead cell stain kit (Thermo Fisher Scientific, Waltham, MA, USA), CD3-BV711 (BD Biosciences, San Jose, CA), CD4-BV650 (BD Biosciences), CD19-PE-Cy5 (BioLegend, San Diego, CA, USA), HLA-DR-APC-CY7 (BioLegend) and, CD45-BV786 (BD Biosciences). For intracellular staining, cells were washed with PBS and incubated for 20 min with cytofix/cytoperm (BD Biosciences) according to manufacturer’s instructions. After fixation, cells were washed with perm wash buffer (BD Biosciences) and incubated for 20 min at RT with perm wash buffer containing CD68-AF488 (BioLegend) and CD206-PE (BD Biosciences) antibodies. Fluorescence minus one (FMO) or unstained cells were used as a control. Stained cells were acquired using the LSRFortessa (BD Biosciences) with FACSDiva^™^ software. Data were analyzed using FlowJo version 10.6.0 (FlowJo, LLC, Ashland, Oregon). LNMCs were identified as CD45^+^/CD3^−^/CD19^−^/HLA-DR^+^ by flow cytometry. Further gating was used to identify specific macrophage phenotypes as described in **(Supplementary Fig.3)**.

#### Lunaphore COMET Multiplex Imaging

Multiplex panels were designed according to project needs for the investigation and characterization of macrophage markers within human LNs. Sequential immunofluorescence multiplex staining was conducted using the COMET, an automated system controlled by its own software. Reagents and antibody volumes were calculated and generated by the COMET control software according to the pre-programmed protocol using the following antibody panel: Ki67 (IR62661, Dako/Agilent Technologies), CD206 (685645 MAB25341, R&D Systems), CD68 (clone KP1, Dako/Agilent Technologies), IBA-1 (ab178846 EPR16588, Abcam), CD163-AF647 (ab218294, EPR14643–36 Abcam), CD3 (M7254, Dako/Agilent Technologies), CD19 (IR656, Dako/Agilent Technologies), CD4-AF647 (ab196147, Abcam), Gagp17 (HRP-20081, NIH) and Gagp24 (MAP1341, Abnova).

Primary and secondary antibodies were diluted in MSB according to their optimized dilution and concentration and stored at 4°C until the protocol was ready to be run. COMET reagents were prepared according to the manufacturer’s protocol and stored in the appropriate locations within the COMET module. Following the placement of the appropriate reagents in their respective places, the protocol was initiated. For more detailed instructions regarding the preparation of COMET reagents, please refer to the COMET manual or to the reference by Najem and colleagues (37).

#### DNAscope *in situ* hybridization (ISH)

DNAscope ISH was conducted using the RNAscope 2.5 HD assay kit (Advanced Cell Diagnostics (ACD), Newark, CA, USA, Cat No: 322300) and the RNAscope multiplex fluorescent kit v2.0 (ACD, Cat No: 323100) as per manufacturer’s instructions. LN FFPE blocks were sectioned into 5μm slices and affixed onto Surgipath X-tra adhesive pre-cleaned microslides in preparation for antibody staining. Briefly, pre-treated samples were hybridized with the HIV-1-CladeC-*gag-pol*-sense (Cat number 444021) probe to detect HIV subtype C DNA at 40 °C for 2 hours. Next, the samples were incubated with signal amplification probes and horseradish peroxidase conjugated secondary antibodies. The signal was detected with Opal fluorophores (AKOYA Biosciences) for the multiplex fluorescent assay. Slides were imaged with the Zeiss Imager Z.2 microscope (TissueGnostics, Vienna, Austria).

#### DNAscope ISH multiplexed with RNAscope *in situ* hybridization ISH

RNAscope ISH was conducted on sectioned LN tissue slides that already underwent the DNAscope protocol as described above. The RNAscope 2.5 HD assay kit (Advanced Cell Diagnostics (ACD), Newark, CA, USA, Cat No: 322300) and the RNAscope multiplex fluorescent kit v2.0 (ACD, Cat No: 323100) were used as per manufacturer’s instructions. Briefly, post-DNAscope samples were rinsed, pre-treated, and hybridized with the HIV-1 *gag-pol* probe (Cat No: 317691) at 40 °C for 2 hours. Next, the samples were incubated with signal amplification probes and horseradish peroxidase-conjugated secondary antibodies. The signal was detected with a different opal fluorophore (AKOYA Biosciences) for the multiplex fluorescent assay. Slides were imaged with Axio Observer and TissueFAXS imaging software (TissueGnostics).

#### Cell sorting and digital droplet PCR (ddPCR)

LNMCs were surface stained with the panel of antibodies including LIVE/DEAD fixable Aqua dead cell stain CD3 BV711, CD4 BV650, CD45 BV786, CD19 PE-CY5 and CD56 APC. Cells were sorted using the FACS Aria Fusion (BD Biosciences). Gating strategies for sorting are detailed in **(Supplementary Fig. 9A)**.

Droplet digital PCR (ddPCR) was used to determine the HIV copy number in myeloid and paired CD4+ T cells from each study participant as previously described(61). DNA was extracted from myeloid and CD4+ T cells using DNeasy Blood & Tissue Kits (QIAGEN). Total HIV-1 DNA and host cell concentrations in the DNA extracts was estimated using primers and probes covering HIV-1 5′ LTR-gag, HXB2 coordinates 684–810, (forward primer 5′−457 TCTCGACGCAGGACTCG-3′, reverse primer 5′-TACTGACGCTCTC GCACC-3′ probe/56–458 FAM/CTCTCTCCT/ZEN/TCTAGCCTC/31ABkFQ/), and human RPP30 gene (forward primer 5′−459 GATTTGGACCTGCGAGCG-3′, reverse primer 5′-GCGG CTGTCTCCACAAGT-3′, probe/56–460 FAM/CTGACCTGA/ZEN/AGGCTCT/31AbkFQ/). Thermocycling conditions were 95°C for 10 min, 45 cycles of 94 °C for 30 s and 60 °C for 1 min, 72 °C for 1 min. The Bio-Rad QX200 Droplet Reader was used to detect positively amplified DNA within droplets and data was analyzed using QX Manager Software v1.2 (Bio-Rad).

#### High dimensional UMAP analysis

For dimensionality reduction and visualization, we employed the multiparametric global Uniform Manifold Approximation and Projection (UMAP) algorithm using R studio (version 2023.09.1+494), due to its effectiveness in revealing underlying structures in high-dimensional data. To identify distinct cell clusters within the UMAP space, we further employed the Flow Self-Organizing Map (FlowSOM) clustering algorithm, and to identify differential LNMC clusters. To further characterize the identified clusters, we utilized the unsupervised hierarchical clustering heatmap to summarize the mean fluorescence intensities of each loaded parameter, whereby warm colors (dark red) indicate high expression and cold colors (dark blue) indicate low expression. The frequency (%) was calculated and compared between cell clusters of interest to assess pro-inflammatory, anti-inflammatory and transitioning macrophage (62, 63). The frequency is calculated as follows:

$$\text{Frequency}\;(\%)=\frac{\text{number of cells in the cluster}}{\text{total number of cells}}\times 100$$

#### Quantitative analysis of LN macrophage frequency, and HIV Gagp24 protein co-expression by TissueQuest

Quantitative image analysis of macrophage frequency and HIV Gagp24 protein in whole tissue section scans was conducted with TissueQuest software (TissueGnostics). Total area measurements and nuclear segmentation analysis were performed on each whole tissue scan. The DAPI channel was used as a master channel for nuclei detection in each whole LN tissue section. Thereafter the grey scale images from each channel were analyzed and quantified according to their expression and reported as a measure of density (cells/mm)^2^ **(Supplementary Fig.2)**.

#### Quantitative analysis of HIV *gag-pol* DNA expression and CD68 co-expression by HALO

Quantitative image analysis of HIV vDNA in LN tissues was performed using the FISH-IF module on HALO software (version v3.6.4134.396; Indica Labs) as previously described (64). Monochrome tiff image files of individual channels were exported from TissueFAXS and uploaded onto HALO where a merged image was formed and used for subsequent quantitative analysis. For the DNAscope analysis, GCs and EF regions which harbored high observed frequencies of vDNA were segmented to form the ROIs for analysis (on average, 1500 cells were segmented per ROI). 3 EF ROIs were segmented per donor and 1–3 GC ROIs were segmented per donor based on the frequency of GCs within the LN tissues. DAPI was used as a master channel to detect and segment all nuclei. Thereafter, thresholds were set to mask the vDNA probe signals and CD68^+^ macrophages harboring vDNA. The frequency of cells expressing HIV DNA was quantified as a proportion of DNA-harboring cells out of the total cells per region. The proportion of vDNA arising from CD68^+^ macrophages was also assessed. The probe scoring system was set to routine parameters to reflect the minimum probe copies per cell summarized as probe score (minimum probe copies per cell): 0+ (0); 1+(1); 2+(4); 3+ (10) and 4+ (16).

The H-score indicates the amount of expression of the probe directed at gag-pol HIV DNA/RNA based on the minimum intensity thresholds. The H-score is calculated based on the equation:

H-Score=(1×%Probe1+cells)+(2×%Probe2+cells)+(3×%Probe3+cells)+(4×%Probe4+cells)

#### Specific Quantification of Inducible HIV-1 by RT-LAMP (SquHIVLa) Assay

Matched LNMCs and peripheral blood mononuclear cells (PBMCs) from PLWH were utilized for the SquHIVLa assay to quantify the frequency of cells expressing tat/rev from multiply spliced RNA (msRNA). PBMCs or LNMCs were first depleted of CD8^+^ T cells using the EasySep^™^ Human CD8 Positive Selection Kit II (Cat. No. 17853; STEMCELL Technologies) according to the manufacturer’s instructions. The remaining unlabeled fraction was subsequently subjected to CD4^+^ T-cell enrichment using the EasySep^™^ Release Human CD4 Positive Selection Kit (Cat. No. 17752; STEMCELL Technologies). The positively selected CD4^+^ cells were collected for downstream assays, and the remaining unlabeled fraction was designated as the myeloid cell population.

SQuHIVLa assays on paired CD4^+^ T cells and myeloid cells were performed as previously described (Hossain, et al., 2024). Briefly, cells were stimulated with 100 ng/mL phorbol 12-myristate 13-acetate (PMA; Sigma-Aldrich) and 1 μg/mL ionomycin (Sigma-Aldrich) for approximately 12 hours. Following stimulation, cells were counted, and serial dilutions were prepared such that each reaction contained the established RT-LAMP SQuHIVLa mastermix and either 20,000, 5,000, 1,250, or 313 cells (24–48 replicates per dilution) for samples obtained from virally suppressed PLWH, or 5,000, 1,250, 313, or 78 cells (24–48 replicates per dilution) for samples obtained from viremic PLWH.

Reactions were incubated at 45 °C for 60 minutes, followed by continuous amplification at 65 °C with fluorescence acquisition every 30 seconds for 90 minutes. Upon completion of the RT-LAMP reaction, positive wells at each dilution were enumerated, and the frequency of cells expressing tat/rev msRNA was calculated using a maximum-likelihood approach. The specificity of SQuHIVLa was confirmed using both CD4^+^ T cells and myeloid cells from PBMC and LNMC samples of PLWoH participants, with no positive signals detected in these negative-control populations

### Statistical analysis

Statistical analysis and graphical presentation were performed using GraphPad Prism version 10.2.0 software (GraphPad Software Inc., La Jolla, CA, USA). The Shapiro-Wilk normality test was used to test for normality. Student’s unpaired t-test was used to compare differences between two unpaired groups with parametric data distribution. For nonparametric data distributions, the Mann-Whitney U test was used to compare differences between two groups. For paired data analysis, the Student’s paired t-test was used for parametrically distributed data, whilst the Wilcoxon sum-ranked test was used for non-parametric data and comparisons between low sample sizes. Spearman’s rank correlation analysis was performed for non-parametric data, and the correlation rank (r) was reported as a measure of the strength of the association. A p-value of <0.05 indicates statistical significance.

### Study approval

All study participants provided written informed consent prior to inclusion in the study. Ethical approval for the study was granted by the University of KwaZulu-Natal Biomedical Research Ethics Committee (protocol number BF298/14) and the Institutional Review Board of Massachusetts General Hospital (protocol number 2015-P001018).

## Supplementary Material

Supplementary Files

This is a list of supplementary files associated with this preprint. Click to download.

- SupplementaryMaterialsWithlegendsPDF.pdf

## Figures and Tables

**Table 1.** Participant demographics and clinical data.

	Gender					
	Female	Male	Age Ranges, (Median)	CD4 Count, mean cells/ L (SD)	HIV plasma viral load (copies/mL)^b^	Days on ART (Median)
**All *n* = 58**	54 (93.1%)	4 (6.9%)	19–61 (23)		-	-
**PLWoH *n* = 19 (32.8%)**	19 (100%)	-	19–25 (22)	794 (369.0)	N/A	N/A
**PLWH *n* = 39 (67.2%)** ^a^	35 (89.7%)	4 (10.3%)	22–28 (26)	682 (391.0)	59–400,000 (2300, 33.3%)	9–3810 (566)
**Acute *n* = 6**	6 (100%)	-	22–28 (25)	979.5 (282.5)	^c^	120–3359 (843)
**Chronic *n* = 27**	24 (88.9%)	3 (10.1%)	19–40 (23)	639.0 (362.0)	59–6000 (475, 37.0%)	9–3810 (180)
**Utx/ Uk *n* = 6**	5 (88.3%)	1 (11.7%)	22–61 (41)	375.0 (664.0)	^d^	-

Data closest to the time of lymph node excision.

a PLWH are classified according to ART status, wherein treatment during Fiebig stage I-V is acute-treated and after Fiebig stage V is chronic-treated.

b Under HIV plasma viral load, the median was calculated for participants with detectable viral load (>20 copies/mL), and the median was followed by % PLWH with detectable viral load.

c Median cannot be determined (only 1/6 participants had a detectable pVL).

d Median cannot be determined (only 2/6 participants had a detectable pVL).

## Data Availability

All relevant data are available within the manuscript and its supporting information files
